# Data on the evaluation of structural index from aeromagnetic enhanced datasets with the application of Euldph-λ semi-automatic algorithm

**DOI:** 10.1016/j.dib.2018.05.090

**Published:** 2018-05-23

**Authors:** John Stephen Kayode, Yusri Yusup

**Affiliations:** Environmental Technology, School of Industrial Technology, Universiti Sains Malaysia, 11800 Pulau-Pinang, Malaysia

## Abstract

A secondary dataset was generated from the Euldph-λ semi-automatic Algorithm, (ESA) developed to automatically computes various depths to the magnetic anomalies using a primary data set from gridded aeromagnetic data obtained in the study area. Euler Deconvolution techniques, (EDT), was adopted in the identification and definition of the magnetic anomaly source rocks in the study area. The aim is to use the straightforward technique to pinpoint magnetic anomalies at a depth which substantiate mineralization potential of the area. The ESA was integrated with the imaging function of Oasis Montaj 2014 source parameter from Geosoft® Inc. From the data, it could be summarized that similar tectonic processes during the deformation and metamorphic activities, the subsurface structures of the study area produce corresponding trending form.

**Specifications Table**

**Table:** 

Subject area	*Earth and Planetary Science*
More specific subject area	*Geophysics*
Type of data	*Excel files and figures*
How data was acquired	*Extracted data from the Airborne survey, Proton Magnetometer Equipment was used for the data acquisition with; a fixed-wing aircraft, 3x Sintrex CS3 Caesium Vapour magnetometer, FASDAS magnetic counter, KING KR 405/KING KR 405B radar altimeter and ENVIRO BARO/DIGIQUARTZ barometric altimeter as the acquisition parameters.*
Data format	*Data Enhancement Filtered and Analyzed*
Experimental factors	*The database filters, i.e., convolution filters processes were first applied to the data to smoothed it and to improve the signal to noise ratio while still preserving the geologic features of interest. To achieve this, some sufficient varieties of geophysical and mathematical filters. Such as the upward continuation, downward integration, and low-pass filters were used to improve the resolution of the aeromagnetic data. The advantages are; (i) to better the output of the maps produce and, (ii) to eliminate the effect of regional data trends. The data helps in the speedy processes that improved gridded datasets through the applications of*
Data source location	*Omu-Aran schist belt, Southwestern, Nigeria, spreading across Latitudes N 7*^*°*^*59׳ 45.6׳׳ and 8*^*°*^*30׳ 22.21׳׳ and Longitudes E 5*^*°*^*0’ 1.55׳׳ and 5*^*°*^*29׳ 57.95׳׳, bounded by the land area of 3381.12 km*^*2*^
Data accessibility	*The data is with this article.*

**Value of the data**

• Depths computation in geophysical prospection is always the best estimator in the identification and definition of the subsurface magnetic anomaly source rocks with least errors.
• The application of data enhancement techniques offered the opportunity of high accuracy in subsurface minerals prospection.
• Studies of this nature are limited in the area, hence, the data offered a better understanding of the subsurface structures emplaced in this area to the geoscientific community.

## 1 Data

**Fig. 1:**
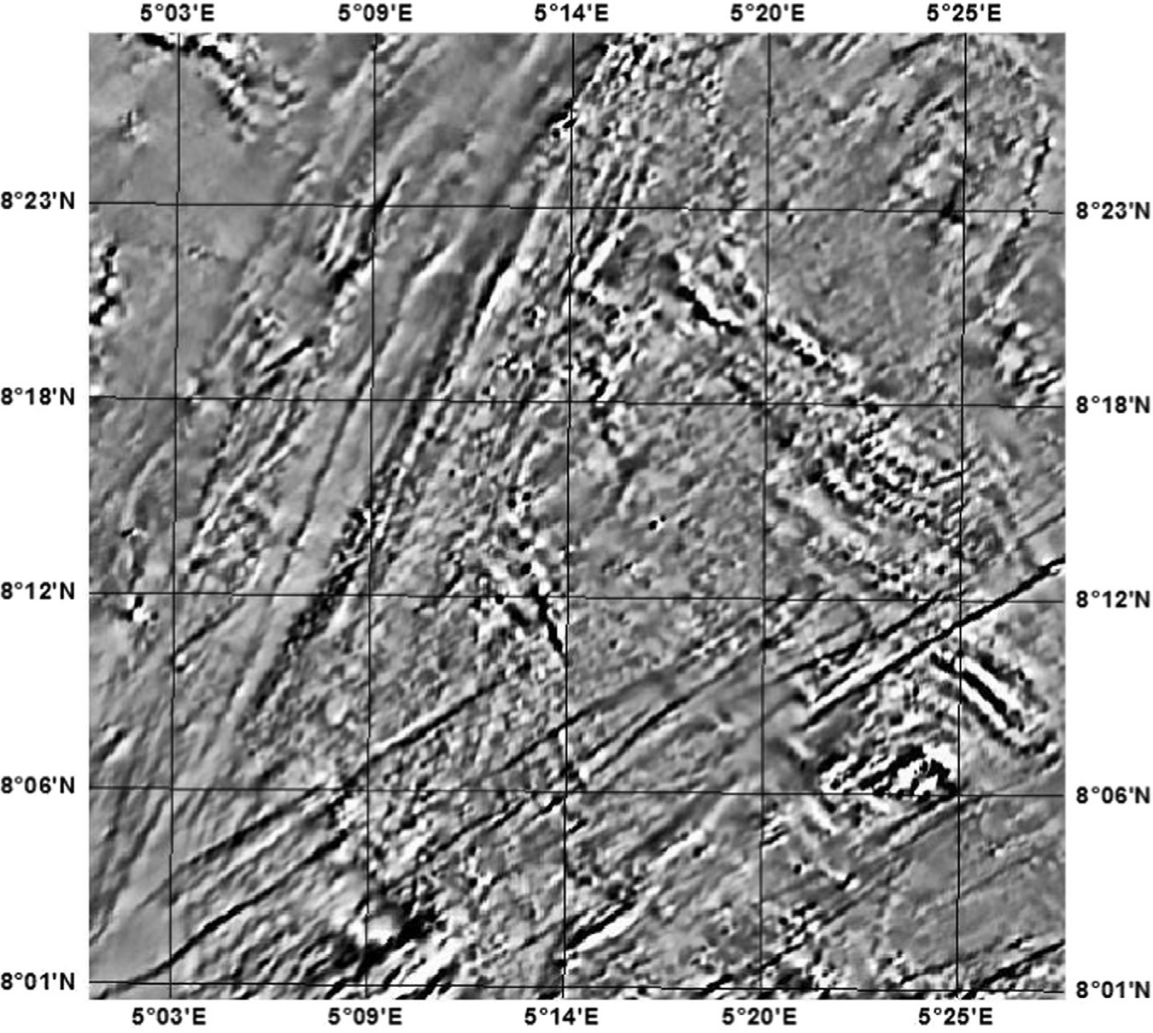
Horizontal derivative map of the study area showing the E-W and N-S cross-section profiles.

**Fig. 2:**
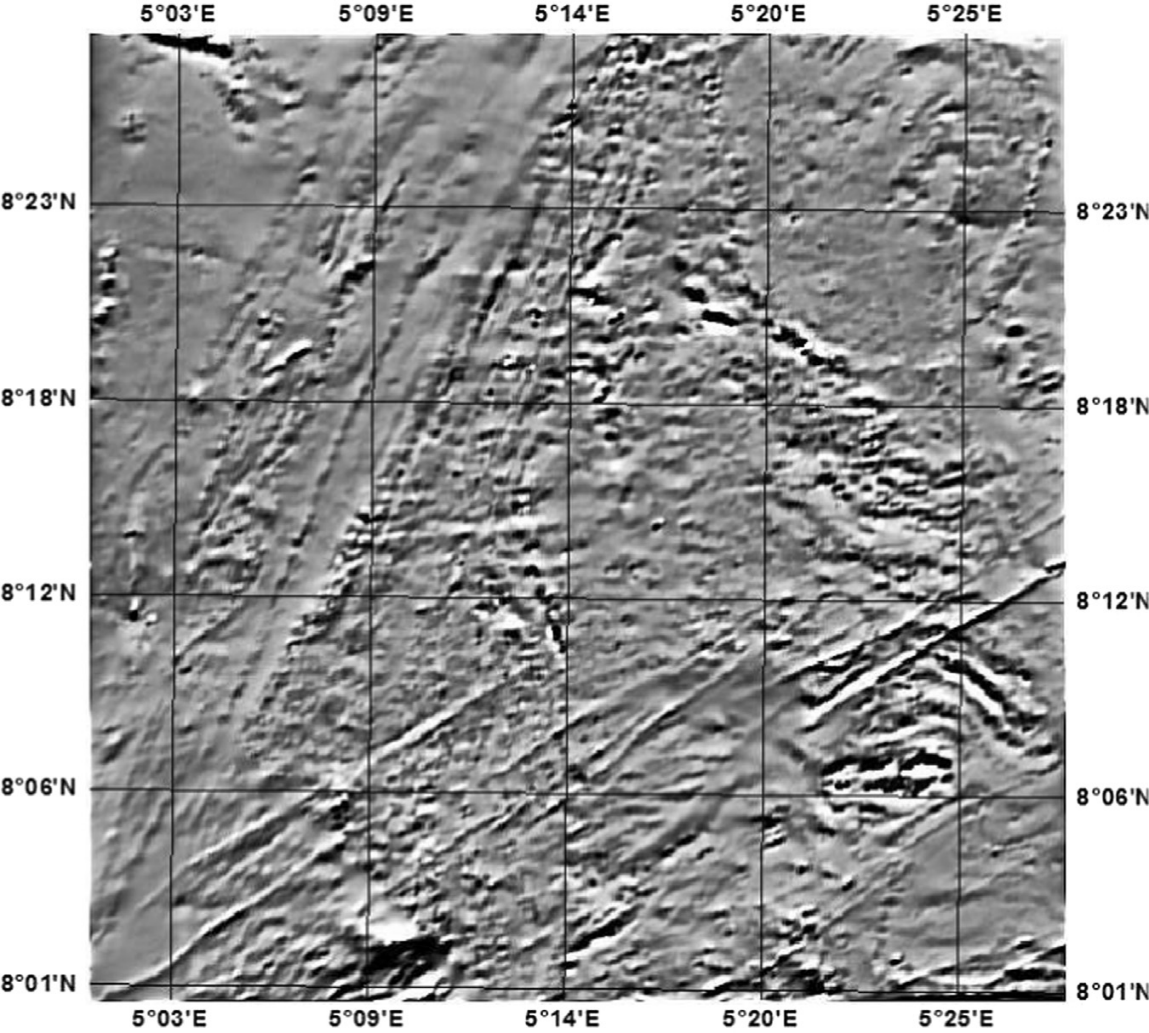
Vertical derivative map of the study area showing the E-W and N-S cross-section profiles.

The high-resolution aeromagnetic data was gridded, using the minimum curvature algorithm of Oasis Montaj 2014 version at a sampling interval of 100 m on a Universal Transverse Mercator (UTM) projection. The gridded data was divided into East-West and North-South geomagnetic cross-section profile lines, as shown in Fig. 1, Fig. 2, covering a total of 343,982 number of primary data points in a block of about 58.7 × 57.6 km [1], [2].

Each sub-grid data was de-trended to eliminate the mean and the trends by taking out the first order surface that best fit the data using the least-squares method. Edges of the sub-grids are narrowed by the applications of a 10-point cosine bell to damp discontinuities at the edges. The zeros were appended beyond the grid edges to obtain sub-grids of 559 by 559 data sampling points which reduced the data to 312,481 secondary data points.

A two-dimensional Discrete Fourier transform, (DFT) was used by the Oasis Montaj software from Geosoft® Inc to estimate each sub-grid. The two-dimensional power spectrum was calculated and simplified to a one-dimensional radial spectrum by averaging values within concentric rings about the spectral origin and normalizing with respect to its value at r = 0 [3].

The depth to the base of the magnetic crust was then determined with the applications of the ESA technique using the average depth to the deepest magnetic horizon and the position of the spectral peak along a profile. The primary aeromagnetic raw datasets and the results from the structural index analysis are as presented in the Supplementary files.

## 2 Experimental design, materials and methods

The primary data exploration involved the initial data processing methods and preparations through the applications of the necessary DFT filtering techniques of data reduction and the applications of the ESA technique to generate data of the depths to the magnetic anomaly sources in the study area. This stage was carried out to identify the best relevant variables, i.e., the depths and Structural Index (SI), values that define the nature of the anomalies. The stage involves the choice of simple shape models that represent the anomalies.

The aeromagnetic data processing was transformed from space domain to frequency domain with the applications of DFT filters. This method made the computation of continuous discrete potential field data less stressful as the speed with which the data was processed is faster than the old method of applying Taylor׳s series [1].

**Fig. 3:**
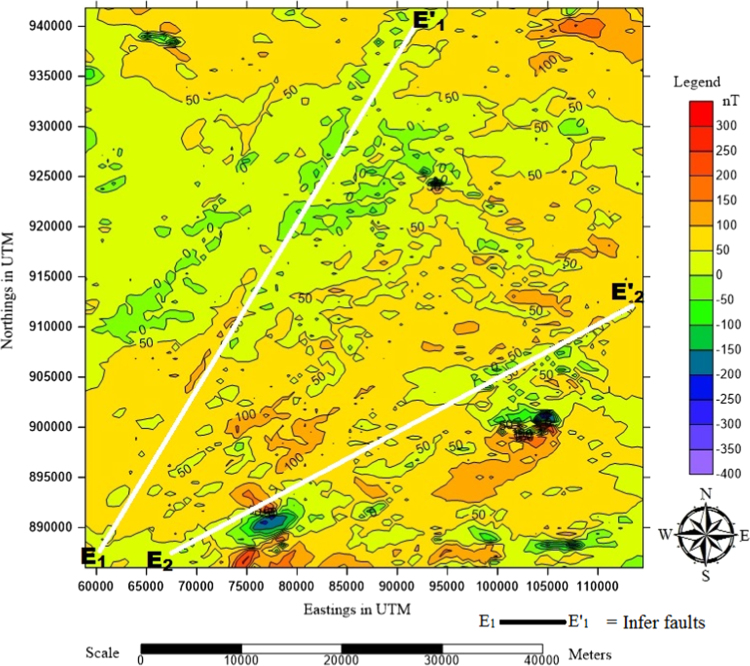
Aeromagnetic data map of the study area showing the major faults E_1_-E_1_’ and E_2_-E_2_’ as marked by the white lines.

In magnetic method of geophysical prospecting, the aeromagnetic data is used to prospect for both magnetic minerals (high magnetic intensity values) and none magnetic minerals (low magnetic intensity values) directly. The method involves tracing of ore-bearing formations and geological features such as faults /fractured (Fig. 3), or rock contacts zones, a dyke, a pipe (horizontal and vertical), a ridge, a cylindrical object, etc. Usually, the raw data collected from the field must be corrected for time variations of the Earth׳s magnetic field and the Aircraft platform motion. In addition to this, the International Geomagnetic Reference Field (IGRF) was used to remove non-crustal effects from the data before processing. Though this process was done by the company that acquired the data for the Nigerian Government, the Oasis Montaj package also performed the task before the applications of other filtering tools [1].

To deviate from the known trial-and-error or indirect determination of source parameters in magnetic data interpretations, the inverse methods involve direct determinations of the magnetic source parameters from the measured data. In magnetic data interpretations, each anomaly has an infinite number of permissible sources. The interpreter needs to narrow down these infinite number of permissible sources to some smaller number by way of constraining the parameters of the source rocks. Even though, there are only two parameter sets that govern the shape of any magnetic anomaly, i.e., the shape of the causative body and the magnetic materials distributions within the body. In magnetic data analysis, one of these two must be fixed or kept constant whilst the other varied. Simple models are constantly used to estimate magnetic source parameters, that is, the depth, strike direction, inclination and declination angles, etc. However, simple pole and dipole approximation are commonly applicable in aeromagnetic anomalies associated with subsurface mineral exploration. Most airborne magnetic interpretations use a vertical prism for basement depth estimation [1].
